# Technical Evolution, Clinical Application, and Outcomes of Endoscopic Transpapillary Gallbladder Drainage in Acute Cholecystitis: A Review

**DOI:** 10.3390/jcm15156007

**Published:** 2026-08-02

**Authors:** Junqi Zhang, Wei Jiang, Yongjun Wang

**Affiliations:** Department of Gastroenterology, Beijing Friendship Hospital, Capital Medical University, Beijing 100050, China; zhangjunqi@mail.ccmu.edu.cn (J.Z.); jfw899w@gmail.com (W.J.)

**Keywords:** acute cholecystitis, endoscopic retrograde cholangiopancreatography, gallbladder disease, gallbladder drainage, transpapillary, endoscopic transpapillary gallbladder drainage, endoscopic nasogallbladder drainage, endoscopic gallbladder stenting, percutaneous transhepatic gallbladder drainage, endoscopic ultrasound-guided gallbladder drainage

## Abstract

**Background:** Acute cholecystitis (AC) is a common inflammatory disease of the gallbladder. Although laparoscopic cholecystectomy remains the standard treatment, gallbladder drainage may be required in patients who are unsuitable for early surgery. Endoscopic transpapillary gallbladder drainage (ETGBD), including endoscopic nasogallbladder drainage (ENGBD) and endoscopic gallbladder stenting (EGBS), has increasingly been used as a minimally invasive internal drainage approach. **Aims:** This review aims to clarify the role of ETGBD in AC by integrating stepwise technical failure analysis with a practical selection framework among other drainage techniques. **Methods:** This narrative review was conducted through a structured literature search of PubMed, Embase, Web of Science, and the Cochrane Library up to 30 April 2026. **Results:** ETGBD preserves native gallbladder anatomy and may have specific value in selected patients, particularly when gallbladder preservation is desirable or concomitant endoscopic retrograde cholangiopancreatography (ERCP) is indicated. Recent technical developments, including cholangioscopy and advanced drainage devices, may support procedural planning and clinical management in selected difficult cases. Compared with percutaneous transhepatic gallbladder drainage (PTGBD) or endoscopic ultrasound-guided gallbladder drainage (EUS-GBD), ETGBD should be regarded as a complementary option rather than a universal substitute, and its application remains limited by variable technical success, cystic duct anatomy, operator expertise, and center experience. However, ETGBD may also be combined with other techniques, providing additional options in selected complex clinical scenarios. **Conclusions:** Adequately powered multicenter comparative studies with standardized outcome definitions are needed to clarify the optimal role, safety, recurrence risk, and long-term stent management of ETGBD.

## 1. Background

Acute cholecystitis (AC) is a common and potentially severe acute abdominal disorder characterized by gallbladder inflammation, which may progress to life-threatening complications if not promptly managed [1]. Laparoscopic cholecystectomy (LC) remains the standard treatment for symptomatic cholelithiasis and acute calculous cholecystitis. However, in patients with organ dysfunction, substantial comorbidities, frailty, or unacceptable anesthetic risk, LC may be associated with increased perioperative morbidity and mortality [2,3].

The 2018 Tokyo Guidelines (TG18) recommend early laparoscopic cholecystectomy (LC) for patients who can tolerate surgery, whereas patients who do not meet the criteria for early LC should receive initial medical treatment and/or gallbladder drainage according to disease severity, response to initial treatment, and surgical risk [4]. For Grade I–II AC, drainage may be considered when the Charlson Comorbidity Index (CCI) and the American Society of Anesthesiologists physical status classification (ASA-PS) suggest that the patient cannot safely withstand early surgery, or when Grade II disease does not respond adequately to initial medical treatment. For Grade III AC, early LC is reserved for highly selected patients with favorable organ system failure, no negative predictive factors, acceptable CCI and ASA-PS scores, and treatment in an advanced center. Otherwise, intensive medical treatment and organ support should be prioritized, and gallbladder drainage should be considered. The intended role of drainage should be defined before treatment. In patients who are not suitable for early LC because of poor general condition, organ dysfunction, uncontrolled sepsis, or anesthesiologic contraindications, gallbladder drainage may be used as initial treatment [2]. When these factors are potentially reversible, delayed LC may be considered after the patient’s overall condition has improved and perioperative risk has decreased. By contrast, long-term or definitive drainage may be considered in patients who remain unsuitable for surgery despite clinical stabilization, particularly those with persistent severe comorbidities, advanced frailty, or unacceptable anesthetic risk [2,5].

Percutaneous transhepatic gallbladder drainage (PTGBD) is a first-line alternative to surgery with a technical success rate over 95%. However, it is unsuitable for patients with bleeding tendencies, massive ascites, or inaccessible anatomical locations. Its external drainage catheter can cause discomfort, limiting long-term use [6]. Endoscopic drainage techniques, including ETGBD and EUS-GBD, are increasingly used. Endoscopic ultrasound (EUS)-guided gallbladder drainage (EUS-GBD) enables direct gallbladder access via transgastric or transduodenal lumen-apposing metal stent (LAMS) placement. Its efficacy is comparable to PTGBD, but widespread adoption remains limited by device availability and the need for advanced EUS and endoscopic retrograde cholangiopancreatography (ERCP) expertise [7]. ETGBD is a drainage technique that involves the placement of a nasobiliary drainage tube or stent during ERCP [8]. One of its key advantages is the use of a natural drainage route, which preserves the normal gastrointestinal anatomy. Studies have reported that ETGBD can be effective across all severity levels of acute cholecystitis [9,10]. Recent studies suggest that ETGBD can achieve technical success rates of approximately 80–95%; however, lower rates within the 60–80% range have also been reported, likely reflecting differences in technical approach, clinical factors, and study scale [11,12,13,14,15,16]. Unlike EUS-GBD, ETGBD primarily requires ERCP expertise, though its broader application is still constrained by procedural difficulties, operator experience, and clinical factors [17,18]. Ongoing research continues to refine ETGBD equipment and procedural techniques [10,19,20,21]. Given its inherent diversity and flexibility, ETGBD may have clinical value as a complementary option for selected patients with gallbladder disease. The current ETGBD literature is heterogeneous, with evidence spanning technical feasibility, device innovation, comparative outcomes, and long-term stent management. Because much of this evidence remains retrospective and operator-dependent, the findings should be interpreted with consideration of selection bias, referral bias, and center-expertise effects. Although the existing literature has provided valuable insights into gallbladder drainage strategies, the specific technical evolution and practical positioning of ETGBD still require further systematic integration. This review is organized around two interrelated themes. First, it provides a stepwise analysis of ETGBD procedural difficulty and technical failure, incorporating recent advances in endoscopic techniques, accessory devices, dedicated stents, and long-term EGBS management. Second, it develops a practical selection framework among ETGBD, PTGBD, and EUS-GBD for patients with acute cholecystitis who are unsuitable for early cholecystectomy, with particular attention to concomitant ERCP indications, cystic duct accessibility, future interval cholecystectomy, and the intended role of drainage as either a bridge to surgery or definitive treatment. By integrating procedural details with clinical decision making, this review aims to clarify the specific role and practical positioning of ETGBD in the management of acute cholecystitis.

## 2. Literature Search Strategy and Study Selection

For this review, we performed a literature search in PubMed, Embase, Web of Science, and the Cochrane Library, with the final search performed on 30 April 2026. The following search terms were used: (“acute cholecystitis” OR “gallbladder drainage” OR “endoscopic transpapillary gallbladder drainage” OR “ETGBD” OR “endoscopic nasogallbladder drainage” OR “ENGBD” OR “endoscopic gallbladder stenting” OR “EGBS” OR “transpapillary gallbladder stenting” OR “percutaneous gallbladder drainage” OR “PTGBD” OR “percutaneous cholecystostomy” OR “endoscopic ultrasound-guided gallbladder drainage” OR “EUS-GBD” OR “cholangioscopy” OR “SpyGlass” OR “cystic duct” OR “gallbladder stent” OR “lumen-apposing metal stent”). All retrieved articles were screened by the authors before exclusion.

We included studies that met the following criteria: (1) studies related to ETGBD, ENGBD, EGBS, PTGBD, or EUS-GBD for acute cholecystitis; (2) studies reporting clinical outcomes, technical outcomes, adverse events, recurrence, long-term stent management, device innovation, procedural troubleshooting, or comparative outcomes; and (3) case reports or technical feasibility reports describing novel ETGBD-related devices, rescue techniques, or uncommon technical scenarios. We excluded (1) articles unrelated to acute cholecystitis or gallbladder drainage; (2) articles without gallbladder intervention; (3) articles lacking relevant clinical or technical information; (4) duplicate studies; (5) articles judged irrelevant after title, abstract, and full-text screening; and (6) non-English articles. Disagreements between the two authors were discussed, and a third author was consulted for resolution when necessary.

The included original studies were summarized in evidence tables. Guidelines, systematic reviews, meta-analyses, and selected background articles were used to support the discussion of disease management, patient selection, safety, and long-term outcomes, but were not necessarily included in the evidence tables. Because this article is a narrative review, no formal meta-analysis or risk-of-bias assessment was performed.

## 3. Classification of ETGBD

In 1984, Kozarek et al. [8] first proposed cystic duct (CD) cannulation via ERCP for the evaluation and treatment of gallbladder disease. Subsequent studies established the clinical feasibility of ETGBD [22,23,24,25]. ETGBD includes endoscopic nasogallbladder drainage (ENGBD) and endoscopic gallbladder stenting (EGBS). ENGBD uses a nasobiliary catheter for drainage, allowing for external irrigation and multiple cholecystographies, but carries risks of dislodgment and discomfort. EGBS employs internal drainage with a double-pigtail catheter, reducing discomfort but limiting repeated rinsing and monitoring [26,27]. ENGBD is preferred for active decompression or close monitoring, while EGBS suits internal drainage and longer-term management [28]. Additionally, patient preference should also be considered after discussion of the benefits and limitations of each approach. The technical and clinical aspects of ETGBD continue to be actively investigated, and representative studies are summarized in Table 1. Detailed evidence extraction for ETGBD-related studies is provided in Supplementary Table S1.

## 4. Procedure and Technical Considerations of ETGBD

**Figure 1:**
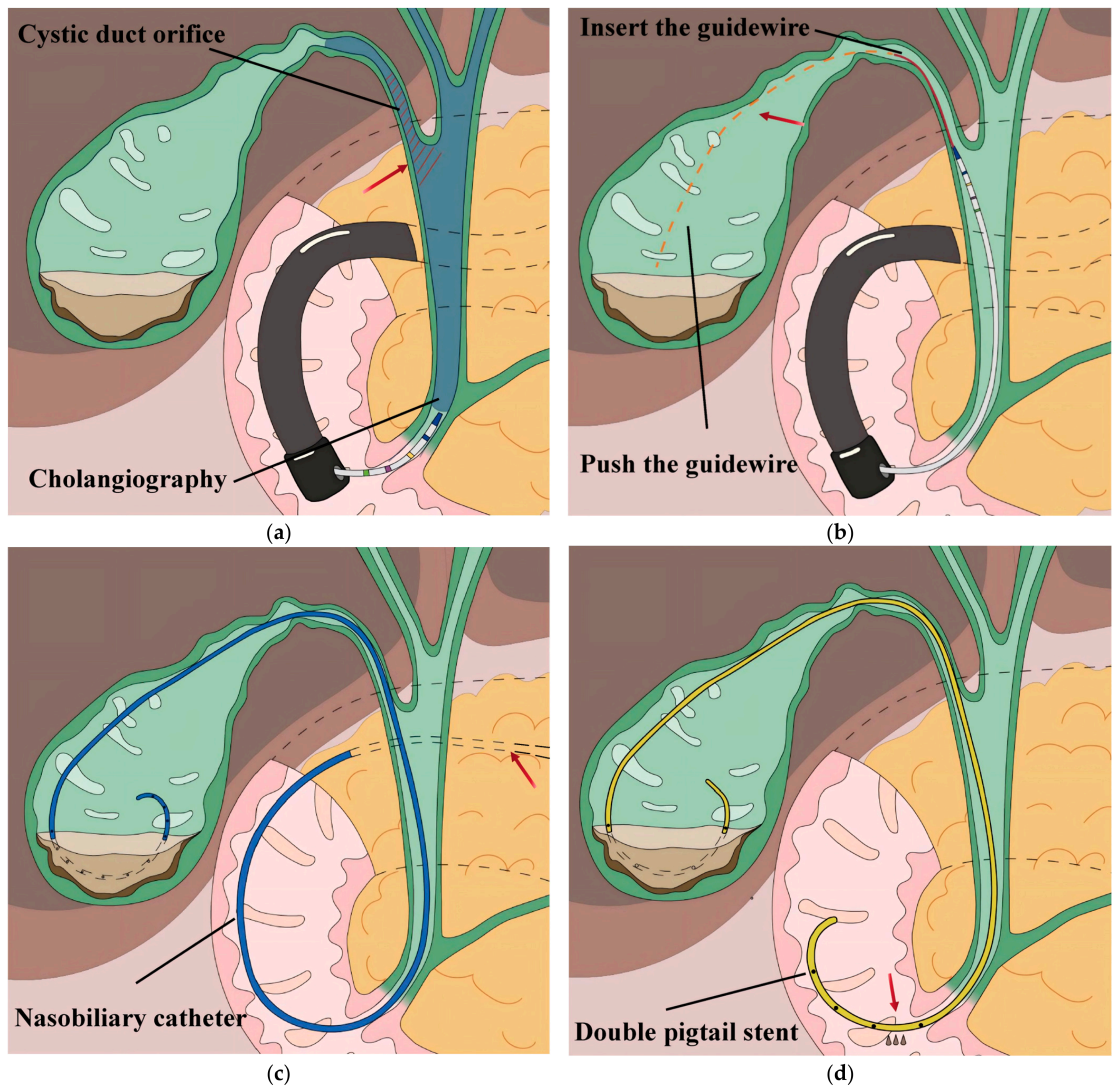
Key steps of the ETGBD process. Black solid and dashed lines indicate superficial and deeper structures, respectively. The blue region represents the cholangiographic area, the red shaded region highlights the cystic duct orifice, and the orange dashed lines indicate the expected device position. (**a**) Cannulate the bile duct and identify the cystic duct orifice; (**b**) insert a guidewire across the cystic duct takeoff and push it into the gallbladder; (**c**) place a pigtail nasobiliary catheter and withdraw the endoscope; (**d**) place a double-pigtail transpapillary stent and withdraw the endoscope. Red arrows highlight the key procedural points and the drainage route.

The ETGBD process involves four key steps: Step 1, cannulating the bile duct; Step 2, inserting a guidewire (GW) into the CD; Step 3, advancing the GW into the gallbladder; Step 4, placing a nasobiliary catheter or stent (Figure 1). Rather than serving as a simple procedural description, this four-step classification provides a practical framework for procedural planning and troubleshooting. It helps localize the point at which technical difficulty occurs, distinguish anatomical barriers from limitations of device or operation, and guide the timely use of adjunctive techniques or alternative drainage strategies. The technical objective, common failure signals, and potential adjunctive strategies for each step are summarized in Table 2.

A primary challenge in Step 1 is visualizing the CD on cholangiography, as severe cholecystitis may obscure both the gallbladder and CD, making cannulation difficult [61]. Limited visualization of the CD increases guidewire failure risk in Steps 2–3, particularly with tortuous anatomy, steep angles, stones, or strictures [19].

Intraductal ultrasonography (IDUS) can assist ETGBD when CD visualization is poor. The use of IDUS was associated with a higher technical success rate, from 76% to 92%, with manageable complications [35]. Pre-procedural PTGBD may facilitate subsequent ETGBD in selected patients by decompressing the inflamed gallbladder and improving cystic duct visualization. Niiya et al. reported that, after PTGBD placement, cystic duct visualization was achieved in 97.1% of patients and subsequent EGBS was successful in 77.1% [20]. In addition, PTGBD-rendezvous rescue has been described after unsuccessful initial EGBS, suggesting that PTGBD may serve not only as temporary decompression but also as a bridge to transpapillary internal drainage in selected cases [32,62]. Therefore, this staged strategy may be considered according to the clinical context when direct ETGBD is technically difficult or when PTGBD has already been performed.

Cholangioscopy, such as the SpyGlass Direct Visualization System (SpyDS), may provide high-definition visualization of the CD and may facilitate ERCP procedures [63]. In a single-center retrospective observational study, Ridtitid et al. attempted conventional EGBS in 104 patients and subsequently performed cholangioscopy-assisted ETGBD in 41 patients after technical failure, increasing the overall technical success rate from 53% to 75% without an apparent increase in AEs or recurrence [37]. Another single-center retrospective study by Yoshida et al. reported similar findings [64]. In an earlier small retrospective series, Shin et al. showed that SpyDS identified cystic duct orifices missed on cholangiography in eight patients, with an 88% technical success rate and a 75% clinical success rate [30]. More recently, Hamamura et al. described a low-cost cholangioscopic method that identified the CD bifurcation when cholangiography was insufficient in a case report [65].

Step 2 can be challenging due to convoluted or stenotic CDs. Commonly used GWs are 0.035-inch and 0.025-inch; smaller or hydrophilic GWs may be needed. Anatomical factors such as proximal and caudal branches, right cranial orientation, and spiral CD shape predict technical failure [19,39]. Suzuki et al. [10] reported that the success rate of ETGBD was significantly higher in patients with a straight or single-bend CD (97.5%) compared to those with two or more bends (71.0%). Sato et al. [51] identified CD injury during the procedure as an independent predictor of failure. Interestingly, Sato et al. [43] subsequently evaluated the accuracy of preoperative CT and MRCP for identifying cystic duct direction and location before ETGBD. The accuracy rates of CT were 58% to 79%, while the accuracy rates of MRCP were 58% to 68%. As a result, the preoperative evaluation of the anatomical structure of the CD is relatively limited.

The balloon occlusion technique is frequently employed to insert a GW into the CD takeoff [66]. This method involves inflation of the occlusion balloon proximal to the CD origin, and subsequent GW advancement into the CD. Additionally, balloon occlusion may serve a dual purpose by creating a sealed compartment within the bile duct lumen, which may improve contrast agent administration during cholangiography [67].

In this step, cholangioscopy helps guide the guidewire through the cystic duct into the gallbladder [68]. The cholangioscope can stabilize the GW at the CD orifice and help negotiate the takeoff angle, facilitating deeper advancement into the CD. Additionally, cholangioscopy may also help identify and clear intraductal obstruction [69].

For calculous cholecystitis, proper sphincterotomy may facilitate GW insertion and can be performed alongside treating common bile duct stones [70]. However, incorrect sphincterotomy may drain bile from the common bile duct, obstructing the duct opening and complicating catheter or GW access to the gallbladder. Takenaka et al. [71] successfully used a loop-forming GW to dislodge gallstones in the CD, creating space for a nasobiliary tube insertion. Taken together, ETGBD success is strongly anatomy-dependent, and pre-procedural imaging alone may not reliably predict real-time cannulation difficulty.

Step 3 failure usually reflects either true CD obstruction or loss of GW support, both of which prevent smooth entry into the gallbladder. CD obstruction can be caused by gallbladder wall thickening due to inflammation, or by CD stones or tumors leading to bile duct dilation. A single-center retrospective study involving 11 patients suggested that shorter gallbladder short-axis length and thinner gallbladder wall thickness may be associated with higher procedural success [72]. The authors of a prospective single-center study of 27 patients further identified gallbladder wall thickness as a predictor of ENGBD procedural difficulty [73]. In a multicenter retrospective study involving 323 patients, Maruta et al. reported a technical success rate of 72.8% and found that cystic duct stones, common bile duct dilation, and cystic duct orientation were associated with technical failure [39]. By contrast, recent case reports have described rotatable catheters or sphincterotomes as rescue tools for selected difficult anatomy, including localized adenomyomatosis, caudal cystic duct branching, or steep angulation [56,58].

A looped GW in the CD often results from anatomical variations, which can hinder stent placement. Straightening the looped GW is a key technical challenge. In a single-center retrospective comparative study, Yoshida et al. applied a three-pillar strategy combining cholangioscopy, a flex-type guidewire, and a 3-Fr microcatheter to address Step 3 challenges. The flexible tip of the guidewire facilitated navigation through tortuous cystic ducts, while the slim 3-Fr microcatheter improved guidewire operability and helped overcome cystic duct obstruction. Compared with classical ETGBD, strategic ETGBD achieved a higher technical success rate (96.9% vs. 72.0%), with similar clinical success (93.7% vs. 91.7%) and adverse event (AE) rates (10.8% vs. 12.0%) [21]. Case reports by Jimbo et al. and Nakahara et al. further suggested that a small-caliber intraductal balloon catheter or a double-guidewire method may help straighten and stabilize a looped guidewire [74,75].

Step 4 involves the placement of a nasobiliary catheter or stent. ENGBD uses 5–7 F pigtail tubes, allowing fluid analysis to guide treatment [76,77]. Pus or blood in the drainage fluid may indicate ongoing infection or bleeding, and saline irrigation may help maintain drainage. For EGBS, the 7 F to 10 F double-pigtail stent is commonly used, but mismatches in stent length, hardness, or shape can lead to migration or cholecystitis recurrence.

Current research on ETGBD devices mainly focuses on improving stent drainage, reducing migration, and facilitating delivery across difficult cystic ducts. Dedicated gallbladder stents have mostly been evaluated in small observational studies. In a single-center retrospective comparative study, the GBest-N stent, which has a three-dimensional spiral tip and side holes, achieved a technical success rate of 100%, comparable to that of conventional stents, while reducing the rate of late adverse events (4.3% vs. 40.4%) and showing no migration, compared with a migration rate of 31.9% for conventional stents [34]. The IYO-stent, another spiral-shaped stent with multiple side holes, achieved technical and clinical success rates of 91% in a small single-center retrospective case series of permanent ETGBD, with no recurrence reported [46]. From a technical perspective, helical or spiral stent designs may help reduce migration, while a smaller 5 F diameter may facilitate insertion in patients with cystic duct stones or complex cystic duct anatomy. Multiple side holes may also improve bile drainage and reduce bile accumulation, thereby lowering the risk of stent occlusion. However, the non-integrated design of some dedicated stents may limit procedural flexibility and warrants further refinement before broader adoption [44].

In contrast, although several device improvements have not yet been evaluated in clinical studies and have only been described in case reports or technical feasibility reports, they may still provide useful technical insight and reference value. Ban et al. reported the use of a screw-tip stent retriever that also functioned as a dilator to overcome inner sheath obstruction within the cystic duct [50]. A tapered endoscopic sheath may assist stent placement into the gallbladder fundus when strictures are difficult to traverse [53]. For complex anatomy or recurrent cholecystitis, over-the-wire stent exchange using a loop cutter may facilitate stent replacement while maintaining access [59]. Drill-type dilators may expand the cystic duct and facilitate safer EGBS placement when device delivery is difficult [55]. Although these reports do not establish general superiority, they illustrate preliminary technical approaches for procedural challenges.

Because smaller transpapillary plastic stents may be prone to occlusion, dual stent placement has been explored to improve drainage durability. In a retrospective case series, Sobani et al. reported the feasibility of dual gallbladder stenting in 21 non-surgical candidates, with both technical and clinical success rates reaching 100%, and recurrent cholecystitis occurring in 1 patient [41]. In their retrospective study, Storm et al. further found that the long-term success rate was higher with dual stent placement than with single stent placement (100% vs. 89%), and that recurrence after single-stent therapy or elective stent removal could be managed by repeat ERCP with the placement of two side-by-side stents [45]. In a multicenter retrospective comparative study, Amin et al. also reported comparable outcomes between dual-stent ETGBD and EUS-GBD, suggesting that dual-stent ETGBD may reduce the recurrence concern associated with single-stent ETGBD [78]. This approach has been proposed to reduce stent occlusion risk and dilate the cystic duct, and index placement of both stents may reduce the need for a second ERCP. However, in cases of severe inflammation or edema, dual stents may aggravate the condition; delaying placement of the second stent for several weeks may help mitigate this issue.

Variation in duodenal papilla–gallbladder distance affects stent selection. Using cut ENGBD tubes as stents may facilitate conversion from initial ENGBD to EGBS after inflammation improves [33,48]. However, this strategy generally requires an additional ERCP, which increases procedural burden, although it may simplify subsequent management in selected patients, and this strategy has also been used successfully in patients undergoing elective cholecystectomy. Several multicenter studies have evaluated the effectiveness of this strategy. For example, Doi et al. [33] achieved a 75% technical success rate with no late adverse events in 40 cholecystitis patients using cut ENGBD tubes as EGBS. Similarly, Maruta et al. [49] reported a 90.5% technical and 100% clinical success rate in 21 acute cholecystitis patients. A subsequent study comparing ENGBD-to-EGBS conversion with simple ENGBD removal as preoperative management found lower late AE rates in the conversion group (6.4% vs. 33.3%) [54]. ENGBD tube cutting can be challenging, and using a loop cutter or external marking may reduce migration risk. In their retrospective comparative study, Takahashi et al. [48] found comparable success rates between cut ENGBD tubes and ready-made stents (92% vs. 100%), highlighting their practicality.

Overall, technical failure of ETGBD may occur at any point in the above procedural sequence. Anatomical factors are usually the primary limitation. The AGA Clinical Practice Update noted that cystic duct occlusion caused by tumors, stones, or uncovered metal biliary stents is a major reason for technical failure of ETGBD [5,79]. Severe inflammation or edema, tortuosity, stenosis, and cystic duct stones may also contribute to failure of cystic duct identification, guidewire advancement, or stent delivery [10,19,39]. In practice, anatomical factors may compromise several procedural steps rather than a single isolated maneuver. Second, technical failure is closely related to operator experience and procedural decision. Even when the cystic duct is identified, successful ETGBD still depends on appropriate guidewire selection, catheter orientation, maintenance of guidewire support, avoidance of cystic duct injury, timely use of rescue techniques, and proper application of adjunctive methods such as intraductal ultrasonography and cholangioscopy-assisted cannulation [35,63,64,65,66]. These adjunctive techniques should be applied selectively according to anatomical difficulty and available expertise, rather than as fixed procedural steps. Third, device limitations may affect both technical success and long-term outcomes. Conventional drainage devices are not always able to accommodate individual anatomical variations and clinical needs. Mismatches in device length, stiffness, diameter, shape, or side-hole position may hinder stent delivery, promote migration or occlusion, and contribute to recurrent cholecystitis [34,46]. This explains why recent device development has focused on improving trackability, facilitating delivery across the cystic duct, reducing migration, maintaining drainage, and simplifying long-term stent management [50,55,60]. Therefore, when ETGBD is considered, the possibility of technical failure should be anticipated, and rescue strategies such as cholangioscopy-assisted cannulation, PTGBD-assisted rendezvous, or alternative drainage should be considered when appropriate.

## 5. Safety of ETGBD

Safety issues related to ETGBD should be considered during both patient selection and follow-up. Because ETGBD is performed through ERCP, ERCP-related adverse events may include post-ERCP pancreatitis, cholangitis, post-sphincterotomy bleeding, and duodenal or periampullary perforation. ETGBD-related adverse events mainly arise from cystic duct manipulation and stent placement, including cystic duct injury or perforation, bile leakage, stent migration, and stent occlusion. When stent exchange or removal is required, repeat ERCP may further increase procedural burden and expose patients to additional ERCP-related risks. According to the American Society for Gastrointestinal Endoscopy (ASGE) symposium, ETGBD-related AEs are generally mild and rarely require extended hospitalization [80,81,82].

Post-ERCP pancreatitis and cholangitis are important ERCP-related AEs after ETGBD. Kim et al. [38] retrospectively reviewed 171 patients with acute calculous cholecystitis. Early AEs, defined as events within 2 weeks after ETGBD, included post-ERCP pancreatitis in nine patients (5.2%) which improved with conservative management or endoscopic hemostasis. Delayed AEs, defined as events occurring more than 2 weeks after ETGBD, included stent migration in one patient (0.6%), recurrent acute calculous cholecystitis in one patient (0.6%), and acute cholangitis in six patients (3.5%). These six cholangitis cases occurred in the high-surgical-risk ETGBD subgroup at 150–399 days after the procedure and were successfully managed by ERCP. Inoue et al. [12] prospectively evaluated endoscopic gallbladder inside-stenting combined with lavage and aspiration in 83 poor surgical candidates with acute calculous cholecystitis. Among early AEs, post-ERCP pancreatitis occurred in two patients (2.4%). During a median follow-up of 603 days, late cholangitis occurred in 3 of 66 patients included in the long-term outcome analysis and was treated by stent removal followed by bile duct stent placement. These findings suggest that adequate gallbladder and biliary drainage, careful papillary manipulation, and timely ERCP-based management of suspected stent dysfunction may help reduce or manage these events.

Bleeding may occur during ETGBD, particularly when endoscopic sphincterotomy is performed or when patients are receiving antithrombotic therapy. In their multicenter study involving 130 cholecystitis patients who were divided into antithrombotic therapy (ATT) and non-ATT groups, Sagami et al. [40] found no significant difference in bleeding complications between the two groups. The ATT group had technical and clinical success rates of 84.3% and 97.1%, respectively, while the non-ATT group achieved 89.4% and 100%, with no bleeding incidents in either group. These findings suggest that ETGBD may be a reasonable option for patients receiving antithrombotic therapy.

Cystic duct injury, perforation, and bile leakage are relatively specific concerns during ETGBD because the guidewire, cannula, catheter, or stent must pass through the cystic duct [77]. Nakahara et al. [47] conducted a retrospective study of 23 cystic duct perforation (CDP) patients during ETGBD, reporting a 43.5% technical success rate and an 87.0% clinical success rate, with 90.7% clinical success in non-CDP cases. No significant differences in clinical success or AE rates were observed between CDP and non-CDP groups. The study highlighted that CDP caused by GWs had a higher technical success rate (53.5%) compared to cannula-induced CDP (25.0%), likely due to the wider perforation caused by cannulas, leading to GW dislocation. In cases of failed ETGBD after CDP, rescue management included bile duct drainage during the same ERCP session, PTGBD, EUS-GBD, surgery, conservative treatment, or repeat ETGBD. Sato et al. [51] similarly reported cystic duct injury in 24 of 242 patients (10%) undergoing ETGBD for acute cholecystitis, with no bile peritonitis observed; cystic duct injury was the only independent predictor of technical failure. These findings suggest that forced advancement of the guidewire or cannula should be avoided, and suspected cystic duct injury or perforation should prompt careful cholangiographic assessment, close monitoring during hospitalization, and timely rescue drainage when bile leakage or inadequate gallbladder decompression is suspected [62,83].

Stent dysfunction, including migration and occlusion, remains a concern during long-term EGBS because it may lead to recurrent cholecystitis, cholangitis, or repeat intervention. Stent migration may occur as gallbladder swelling subsides after inflammation improves, whereas occlusion may result from viscous bile, sludge, food reflux, or duodenobiliary reflux [84]. In pooled analyses, stent occlusion, stent migration, and recurrent cholecystitis occurred in 0.39%, 1.30%, and 1.48% of cases, respectively, although these outcomes may vary with patient population, stent type, and follow-up duration [85]. Dedicated stents such as the GBest-N stent, IYO-stent, and integrated delivery plastic stent may help reduce migration, improve drainage, or facilitate placement, but their role should be considered in relation to long-term follow-up data [34,46,60].

Recurrent cholecystitis is a common late clinical issue after gallbladder drainage and is mainly related to persistent gallstones, cystic duct obstruction, incomplete gallbladder decompression, and stent-related dysfunction [86]. A meta-analysis reported an overall recurrence rate of approximately 1.5% after ETGBD, whereas recurrence during long-term EGBS has shown a wider range of approximately 5.0–19.2% [87,88]. In patients initially managed with ENGBD, conversion to EGBS has been associated with a lower recurrence rate than simple tube removal (5.0% vs. 16.0%). Therefore, recurrence after ETGBD should be assessed in relation to cystic duct patency, stent function, and the intended role of drainage. In addition, repeat ERCP may be required for ENGBD-to-EGBS conversion, stent exchange or removal, or staged placement of additional gallbladder stents. Even when not counted as independent AEs, these procedures may increase the procedural burden and re-expose patients to ERCP-related risks [33,41,48,49,54]. Therefore, the need for additional ERCP should be considered when evaluating the long-term safety and practicality of ETGBD.

## 6. Comparison of ETGBD with Other Drainage Methods

### 6.1. Comparison with Percutaneous Transhepatic Gallbladder Drainage

Although PTGBD is effective, it may be unsuitable in patients with ascites or coagulopathy and often causes discomfort. ETGBD is technically more demanding but may offer internal drainage with comparable efficacy, shorter hospitalization, and fewer complications in selected patients [89] (Table 3). Single-center retrospective studies reported that ETGBD could achieve clinical outcomes comparable to PTGBD and might be associated with shorter hospitalization in selected patients [89,90]. A multicenter study further reported similar or more favorable short-term clinical outcomes for endoscopic drainage compared with percutaneous drainage [91]. In a prospective randomized study, Mu et al. observed more favorable perioperative outcomes with ENGBD than with PTGBD, including abdominal drainage tube placement rates, gallbladder pathology, and bleeding [92]. Furthermore, a large-scale analysis by Ali et al. also suggested that ETGBD was associated with significantly lower 30-day inpatient mortality than PTGBD [93]. Therefore, ETGBD should not be viewed as a universal replacement for PTGBD, but rather as an internal drainage option with specific advantages in patients with suitable anatomy and in those expected to undergo subsequent cholecystectomy.

### 6.2. Comparison with Endoscopic Ultrasound-Guided Gallbladder Drainage

EUS-GBD, an internal fistula approach using LAMS, avoids external tubes and supports long-term stent placement [100]. Its saddle-shaped design with flared ends and wide lumen may reduce risks like migration and bile leakage [96]. Unlike EUS-GBD, ETGBD preserves the native gastrointestinal anatomy and avoids creation of a transmural fistula (Table 3). Retrospective comparative studies generally suggest that EUS-GBD achieves higher technical and clinical success rates, shorter procedure times, and lower recurrence rates than ETGBD [95,97,101,102]. However, Amin et al. reported that dual-stent ETGBD achieved outcomes comparable to EUS-GBD in a multicenter retrospective comparative study, with technical success of 86.3% vs. 87.5%, clinical success of 100% vs. 98.1%, similar AE rates (0% vs. 2.0%), and low recurrence rates (5.3% vs. 8.2%) [78]. Inoue et al. [98] found that recurrence of calculous cholecystitis and early AE rates were comparable between EUS-GBD and ETGBD, whereas late AEs, particularly non-cholecystitis events, were more frequent after ETGBD. However, EUS-GBD requires advanced therapeutic EUS expertise and dedicated devices, may increase device costs, and raises concerns regarding future cholecystectomy and LAMS placement [103,104]. Despite lower overall success rates, ETGBD may still be considered in patients with concomitant common bile duct stones because it allows simultaneous ERCP-based stone extraction, and in those scheduled for cholecystectomy because it preserves gallbladder anatomy [38]. EUS-GBD may be better suited for non-surgical candidates requiring permanent drainage [105].

An international multicenter retrospective comparative cohort study reported that PTGBD and EUS-GBD showed higher technical (98% vs. 94%) and clinical (97% vs. 90%) success than ETGBD (88% vs. 80%), though EUS-GBD had a higher procedural AE rate, while PTGBD had more long-term AEs and more complications [82]. Ridtitid et al. [99] compared PTGBD, EUS-GBD, and ETGBD in acute cholecystitis patients with or without common bile duct stones in a retrospective comparative study. For those with stones, PTGBD and EUS-GBD had similar technical success rates (100% vs. 100%), outperforming ETGBD (73.2%). Clinically, ETGBD and EUS-GBD were comparable (96.2% vs. 100%) and superior to PTGBD (87.5%). For patients without stones, PTGBD and EUS-GBD again matched technically (100% vs. 100%), exceeding ETGBD (77.3%), while ETGBD and EUS-GBD achieved higher clinical success rates than PTGBD (94.1% and 100% vs. 63.0%). From a cost-effectiveness perspective, a 3-month cost-effectiveness model suggested that ETGBD may be cost-saving compared with PTGBD ($40,086 vs. $53,712) and economically favored over EUS-GBD, which required an additional $17,722 to avert 1.98 additional hospitalization days, although these findings depended on assumptions regarding hospital stay, LAMS cost, and expert-center performance [106]. Across comparative studies, ETGBD generally has lower technical success rates than PTGBD and EUS-GBD, which could be partly attributable to difficulties in identifying or traversing the cystic duct, dependence on favorable anatomy, and the need for adequate ERCP expertise. Therefore, findings from expert centers may be less applicable to broader clinical practice. Beyond general risks of gallbladder drainage, including bleeding, infection, bile leakage, perforation, and the need for additional intervention, each approach has its own limitations. PTGBD may cause discomfort from the external tube, tube dislodgement, skin infection, and difficulties with catheter care. EUS-GBD carries risks related to transmural puncture and stent placement, such as bile peritonitis, sepsis, stent migration, and stent dysfunction. ETGBD carries risks including post-ERCP pancreatitis, cholangitis, cystic duct injury, perforation, and repeat ERCP for stent exchange or removal [107]. The choice of drainage method in AC should be individualized according to clinical severity, overall surgical fitness, local expertise, and the anticipated need for future intervention. The intended role of drainage should also be defined before treatment. In patients who are temporarily unsuitable for early LC, gallbladder drainage may serve as a bridge to delayed cholecystectomy [108]. In this setting, surgical candidacy should be reassessed after clinical improvement, resolution of sepsis or organ dysfunction, and optimization of comorbidities and anesthetic risk. By contrast, definitive drainage may be considered for patients who remain poor surgical candidates despite clinical stabilization, such as those with persistent severe comorbidities, frailty, or unacceptable anesthetic risk [2,109]. Comparative studies evaluating ETGBD against PTGBD or EUS-GBD are summarized in Table 3, and the full evidence extraction is provided in Supplementary Table S2.

### 6.3. Practical Framework for Drainage Selection

**Figure 2:**
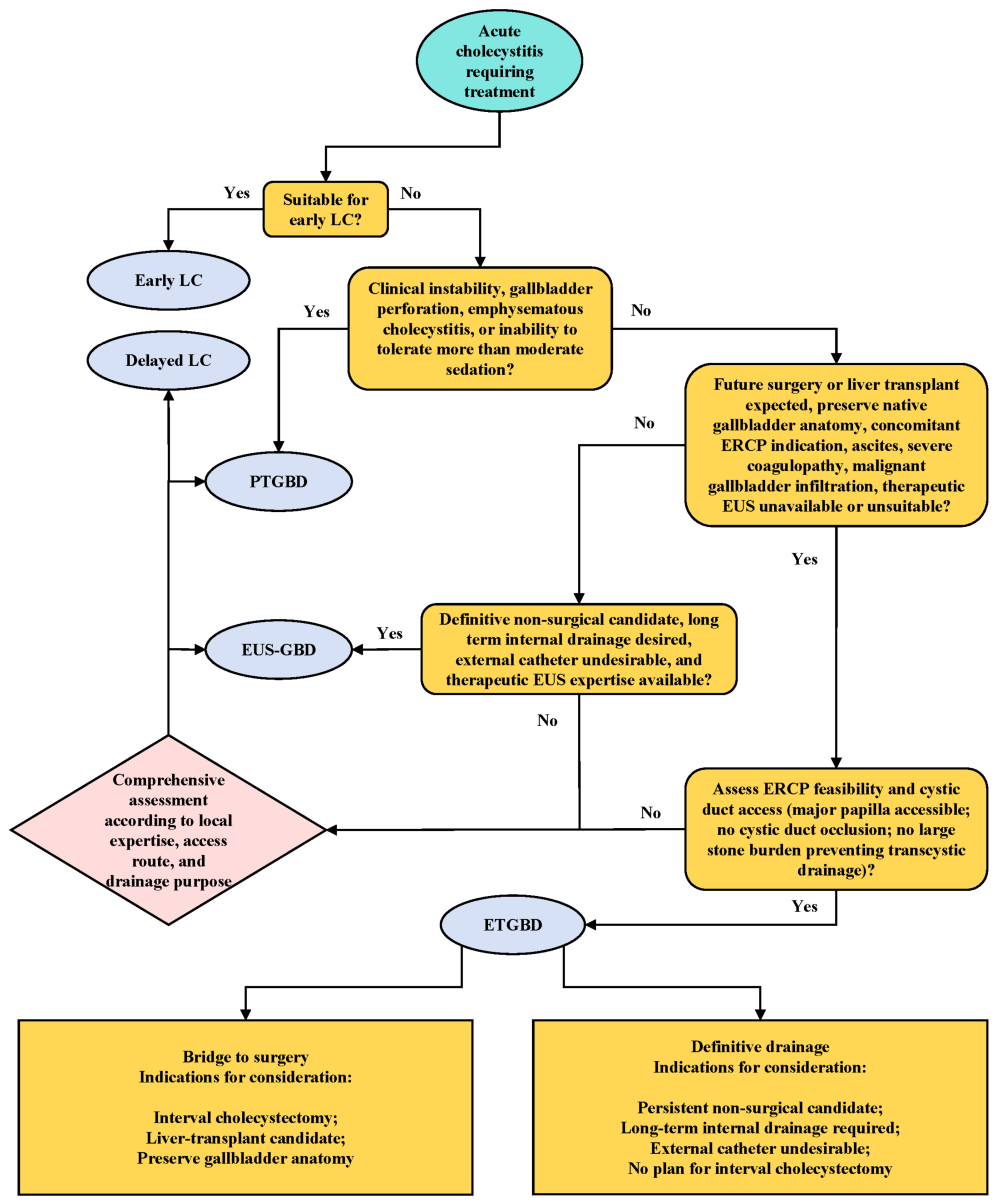
Practical framework for drainage selection. AC, acute cholecystitis; LC, laparoscopic cholecystectomy; PTGBD, percutaneous transhepatic gallbladder drainage; ETGBD, endoscopic transpapillary gallbladder drainage; EUS-GBD, endoscopic ultrasound-guided gallbladder drainage; ERCP, endoscopic retrograde cholangiopancreatography; EUS, endoscopic ultrasound.

Based on the risk-stratified management principles of TG18 and the recent recommendations of the American Society for Gastrointestinal Endoscopy (ASGE) on therapeutic EUS, this framework is intended to guide the individualized management of patients with acute cholecystitis who are unsuitable for early cholecystectomy [4,110] (Figure 2). The optimal drainage strategy should be determined according to disease severity, clinical stability, surgical candidacy, anatomical feasibility, concomitant indications for ERCP, bleeding risk, sedation tolerance, local availability of interventional radiology and therapeutic EUS expertise, and the intended role of drainage as either definitive treatment or a bridge to interval cholecystectomy. 

PTGBD remains an appropriate option when rapid decompression is required, when endoscopic intervention is unsafe or infeasible, or when percutaneous access is safe and readily available, particularly in patients with perforation, emphysematous or gangrenous cholecystitis, clinical instability, or poor sedation tolerance. By contrast, EUS-GBD may be considered when definitive internal drainage is required and appropriate expertise is available. The ASGE guideline suggests EUS-GBD over ETGBD in non-surgical patients, particularly when the major papilla cannot be accessed, when an indwelling metal biliary stent occludes the cystic duct, or when a large gallstone burden makes transpapillary drainage difficult [110]. ETGBD should be regarded as a complementary option rather than a universal substitute for PTGBD or EUS-GBD. It may be particularly considered when gallbladder drainage can be integrated into an already indicated ERCP procedure, such as in patients with concomitant common bile duct stones, cholangitis, or other transpapillary biliary indications. It may also be considered in patients who may subsequently undergo interval cholecystectomy, because it preserves the native anatomical structure. This patient-selection logic is supported by comparative studies. Ridtitid et al. suggested that concomitant common bile duct stones should be considered in drainage selection, because they may favor an ERCP-based transpapillary approach when anatomically feasible [99]. Amin et al. adopted a pragmatic strategy in which ETGBD was attempted in patients who might later become surgical candidates, whereas EUS-GBD was selected for patients who were unlikely to undergo future cholecystectomy [78]. Together, these findings suggest that patient selection should integrate surgical candidacy, the intended role of drainage, transpapillary feasibility, concomitant ERCP indications, and local therapeutic EUS expertise. Therefore, the final choice should be based on multidisciplinary and patient-centered assessment rather than on a single universal rule.

## 7. Combination of ETGBD and Other Technologies

ETGBD is minimally invasive and preserves anatomical structures and mucosa. Its versatility may make it useful in selected complex cases when other approaches are unsuitable or unsuccessful. Although AEs may occur, ETGBD can be considered in selected patients, with alternative drainage strategies available if technical success is not achieved.

Early LC in AC may be complicated by adhesions in Calot’s triangle, increasing the risk of biliary and vascular injury. ENGBD before LC may reduce inflammation and improve cystic duct identification. Toyota et al. [29] reported no conversion to open surgery in 19 ENGBD patients, compared with 11.1% in controls. The ENGBD catheter fixation technique simplifies CD identification, enabling safer minimally invasive procedures.

Self-expanding metallic stents (SEMSs) are widely used in biliary management, including gallbladder drainage, though they may induce cholecystitis [111]. ETGBD combined with SEMS may reduce cholecystitis risk. Ishii et al. [52] reported an incidence of 4.2% with EGBS versus 21.4% in controls. Nakahara et al. [36] reported ETGBD success rates of 83.3% technical and 90% clinical post-SEMS, with 75% success in distal and 100% in perihilar ducts, without major complications. These findings suggest that ETGBD may have a potential role in selected patients with cholecystitis after SEMS placement. Innovatively, Gutkin et al. [112] employed SpyDS-assisted fully covered SEMSs for chronic cholecystitis, facilitating gallbladder drainage and stone removal, highlighting the synergy of endoscopic and stent-based approaches in complex biliary conditions.

A stepwise drainage strategy may be feasible in selected patients. Widmer et al. [31] proposed EGBS via ERCP first, followed by EUS-GBD for failures, and PTGBD if endoscopic drainage is unsuccessful. Among 128 patients, EGBS succeeded in 91%, and the remaining 11 received EUS-GBD, achieving 100% success and symptom improvement. This approach suggests technical feasibility in selected high-risk patients. Iino et al. [113] reported ENGBD followed by EGBS for perforated emphysematous cholecystitis with localized peritonitis, in which PTGBD was considered unfavorable because of potential bile leakage and poor ultrasonographic visualization caused by pericholecystic gas.

## 8. Long-Term Management Following ETGBD

Prolonged stent retention may lead to cholestasis, duodenobiliary reflux, cholangitis, and, rarely, gallbladder perforation or penetration. Therefore, indefinite stent retention may not be optimal, and scheduled exchange or removal should be considered. Permanent EGBS may reduce recurrence in high-risk patients, but optimal exchange intervals and removal timing are unclear.

Prevention of recurrent cholecystitis is a major rationale for long-term EGBS in patients who remain unsuitable for cholecystectomy. Persistent internal drainage was generally associated with lower recurrence than tube removal or observation. Inoue et al. reported no recurrent cholecystitis in 33 patients treated with long-term EGBS during a median follow-up of 473 days, whereas recurrence occurred in 5 of 29 patients (17.2%) managed by observation after percutaneous drainage tube removal [62]. Similarly, Maruta et al. reported a lower recurrence rate after permanent EGBS than after drainage tube removal, with recurrence rates of 5.0% and 16.0%, respectively, during median follow-up periods of 375 and 307 days [42]. In a larger multicenter cohort, recurrent cholecystitis occurred in 10 of 158 EGBS patients (6.3%) during a mean follow-up of 1115.5 days, compared with 23 of 120 patients (19.2%) after PTGBD tube removal [94]. Long-term EGBS may reduce the risk of recurrent cholecystitis by maintaining gallbladder drainage, preserving biliary patency, and providing a “wicking” action. However, the risk of recurrence should still be assessed in relation to patient background, stent strategy, and follow-up duration [32,57,78].

Stent-related dysfunction is another concern during long-term EGBS. Inoue et al. reported late stent-related events in 3 of 33 patients, including stent migration at 70 days and cholangitis at 21 and 440 days after treatment; all patients improved after stent exchange, and one difficult case required PTGBD-rendezvous and guidewire-assisted exchange [62]. As shown by Nakahara et al., the distal migration rate of stents was 31.9%, with migration-related recurrent cholecystitis occurring in 8.5% of patients, whereas no migration or recurrence was observed in the GBest-N group during a shorter follow-up period [34].

Reintervention may be required during long-term EGBS management, either for the treatment of late complications or for planned optimization of drainage. Recurrent cholecystitis, stent migration, and other stent-related adverse events can be managed by stent exchange. In cases of cholangitis and other conditions, the gallbladder stent may be removed first, followed by bile duct stent placement. In technically difficult cases, PTGBD-rendezvous may also be required, with guidewire-assisted stent exchange [12,62]. Reintervention may also be incorporated as part of a planned long-term treatment strategy [41,78]. Storm et al. evaluated long-term transpapillary gallbladder stenting and compared single-stent and double-stent approaches. Patients with only one stent were generally advised to undergo repeat ERCP 1–2 months after the procedure for stent exchange and attempted side-by-side placement of two stents, whereas patients with two stents in place generally did not require routine stent exchange if they remained asymptomatic. In that study, the long-term success rate was higher in the double-stent group than in the single-stent group (100% vs. 89%), and no recurrent acute cholecystitis occurred while two stents were maintained in situ [45]. However, the optimal interval for stent exchange or removal requires further investigation before standardized recommendations can be made.

## 9. Conclusions

ETGBD is a technically demanding and anatomy-dependent internal drainage option for patients with acute cholecystitis who are unsuitable for early cholecystectomy. Current evidence suggests that ETGBD may have clinical value in carefully selected patients, particularly when ERCP is otherwise indicated, such as in patients with concomitant common bile duct stones or cholangitis, or when preservation of native gallbladder anatomy is desirable because interval cholecystectomy remains possible. However, its use should be balanced against its lower technical success rate compared with PTGBD or EUS-GBD, dependence on cystic duct access, need for adequate ERCP expertise, and potential ERCP-related or stent-related adverse events. Drainage selection should therefore be individualized. The available evidence remains predominantly retrospective, heterogeneous, and largely derived from experienced centers; therefore, the current conclusions should be interpreted cautiously. Adequately powered multicenter comparative studies with standardized definitions of technical success, clinical success, adverse events, recurrence, and long-term stent management are needed to clarify the optimal role of ETGBD alongside PTGBD and EUS-GBD.

## Figures and Tables

**Table 1:** Patient and study characteristics of ETGBD.

Author	Year	Format	Scale	Country	Disease	Patients	Drainage Procedure	Drainage Device	Technical Success	Clinical Success	Adverse Events	Recurrence/Late Outcomes	Follow-Up Duration	Technology Application
Kalloo et al. [24]	1994	Retrospective	Unicenter	USA	Symptomatic gallbladder diseases	4	EGBS	Nasobiliary pigtail catheter	100% (4/4)	100% (4/4)	0% (0/4)	0% (0/4)	11–17 mo	Divide the nasobiliary pigtail catheter with laser after successful placement, and leave a length of 1 to 2 cm projecting from the papilla
Toyota et al. [29]	2006	Retrospective	Unicenter	Japan	Acute cholecystitis	22	ENGBD	Nasobiliary drainage tube	81.8% (18/22)		0% (0/18)			Perform ENGBD with LC
Shin et al. [30]	2012	Retrospective	Unicenter	Korea	Acute cholecystitis combined with cholangitis or choledocholithiasis	8	ENGBD	Nasobiliary pigtail catheter	87.5% (7/8)	75.0% (6/8)	12.5% (1/8)		Median 103.5 d (10–315)	Perform ENGBD with the assistance of Spyglass
Widmer et al. [31]	2015	Prospective	Multicenter	USA	Acute cholecystitis	139	EGBS	Double-pigtail stent	91% (117/128)	92% (128/139)	8% (11/139)		Mean 32 mo (1–87)	Perform EGBS or perform EUS-GBS after unsuccessful EGBS
Hatanaka et al. [32]	2016	Retrospective	Unicenter	Japan	Acute cholecystitis	22	EGBS	Double-pigtail stent	77.2% (17/22)	100% (17/17)	0% (0/22)	15.7% (3/19)	Median 229 d (14–880)	Perform gallbladder drainage with a nasobiliary drainage tube and replace the tube with a stent
Doi et al. [33]	2018	Prospective	Multicenter	Japan	Acute cholecystitis	40	EGBS	Nasobiliary drainage tube	75.0% (30/40)	96.6% (29/30)	5.0% (2/40)	0% (0/40)	Median 42 d (12–138) to surgery	Perform gallbladder drainage with a nasobiliary drainage tube and replace the tube with a stent
Nakahara et al. [34]	2019	Retrospective	Unicenter	Japan	Acute cholecystitis	70	EGBS	Stent with tip of a three-dimensional spiral-shaped structure/Pigtail stent	100% (70/70)	100% (23/23) novel; 95.7% (45/47) control	17.4% (4/23) novel; 57.4% (27/47) control	0% (0/23) novel; 8.5% (4/47) control	120 ± 143 d novel; 162 ± 252 d control	Novel gallbladder stent
Sagami et al. [35]	2020	Retrospective	Unicenter	Japan	Acute cholecystitis	100	EGBS	Pigtail stent	76.0% (32/42) without IDUS; 92.0% (46/50) with IDUS	96.0% (79/82) overall	0% (0/42) without IDUS; 6.0% (3/50) with IDUS	3.7% (3/82) early; 7.3% (6/82) late	Mean 176 d (55–337) for late dysfunction	Perform ETGBD with or without IDUS
Nakahara et al. [36]	2020	Retrospective	Unicenter	Japan	Acute cholecystitis after biliary self-expandable metal stent placement	12	ENGBD/EGBS	Nasobiliary drainage tube/Pigtail stent	83.3% (10/12)	90.0% (9/10)	16.7% (2/12)			Remove or retain the SEMS before the ETGBD
Ridtitid et al. [37]	2020	Retrospective	Unicenter	Thailand	Acute cholecystitis	104	EGBS	Double-pigtail stent	53% (55/104) fluoro alone; 75% (78/104) after SOC	100% (78/78)	2% (1/41) SOC; 11% (7/63) fluoro	7% (3/41) SOC; 4% (3/63) fluoro	Median 57 d (1–2405)	Perform EGBS/perform additional SOC-assisted EGBS after unsuccessful EGBS
Kim et al. [38]	2020	Retrospective	Unicenter	Korea	Acute calculous cholecystitis	171	ENGBD/EGBS	Nasobiliary drainage tube/Pigtail stent	90.6% (155/171)	90.1% (154/171)	early 7.6% (13/171); late 4.7% (8/171)	1.4% (1/70)	Mean 495.8 d (43–1117) high-risk EGBS	SpyDS-assisted CD cannulation
Maruta et al. [39]	2020	Retrospective	Multicenter	Japan	Acute cholecystitis	323	EGBS	Nasobiliary drainage tube	72.8% (235/323)	96.2% (226/235)	5.9% (19/323)			Perform gallbladder lavage with a nasobiliary drainage tube, then cut the tube to optimal length and place as a stent
Sagami et al. [40]	2020	Retrospective	Multicenter	Japan	Acute cholecystitis receiving antithrombotic therapy	130	EGBS	Pigtail stent	84.3% (70/83) ATT; 89.4% (42/47) non-ATT	97.1% (68/70) ATT; 100% (42/42) non-ATT	Early 3.1% (4/130); late 1.8% (2/110)	10.9% (12/110)	12-mo patency	
Sobani et al. [41]	2021	Retrospective	Unicenter	USA	Acute cholecystitis	21	EGBS	Two gallbladder stents	100% (21/21)	100% (21/21)	9.52% (2/21)	4.76% (1/21)	Mean 471.74 ± 345.64 d; median 341 d	Dual gallbladder stenting
Maruta et al. [42]	2021	Retrospective	Multicenter	Japan	Acute cholecystitis	71	EGBS	Nasobiliary drainage tube	78.9% (56/71)	94.6% (53/56)	Early 4.2% (3/71); late 5.0% (2/40)	5.0% (2/40)	Median 375 d (IQR 155–710.2) EGBS	Perform gallbladder lavage with a nasobiliary drainage tube, then cut the tube to optimal length and place as a stent
Sato et al. [43]	2021	Retrospective	Unicenter	Japan	Acute cholecystitis	145	ENGBD/EGBS	Plastic stent or nasobiliary drainage tube	87.6% (127/145)		11.0% (16/145) CD injury			
Takano et al. [44]	2021	Case report		Japan	Acute cholecystitis with Billroth-II reconstruction	1	EGBS	IYO-stent	100% (1/1)	100% (1/1)	0% (0/1)	0% (0/1)	6 mo	Single-balloon enteroscopy-assisted ETGBD
Storm et al. [45]	2021	Retrospective	Unicenter	USA	Acute cholecystitis	51	EGBS	Pigtail stent	96.1% (49/51)	100% (49/49)	5.9% (3/51)	4.1% (2/49)	Mean 453 d (18–1879)	Perform EGBS with 1 or 2 stents
Takano et al. [46]	2022	Retrospective	Unicenter	Japan	Acute cholecystitis	11	EGBS	IYO-stent	91% (10/11)	91% (10/11)	Early 9.1% (1/11); late 20.0% (2/10)	0% (0/10)	Median 312 d (109–742)	IYO-stent permanent drainage
Nakahara et al. [47]	2022	Retrospective	Unicenter	Japan	Acute cholecystitis	249	ENGBD/EGBS	Nasobiliary drainage tube/Plastic stent	43.5% (10/23) CDP; 91.2% (206/226) non-CDP	87.0% (20/23) CDP; 90.7% (205/226) non-CDP	CDP 9.2% (23/249); ERCP-related AE 5.6% (14/249)			
Takahashi et al. [48]	2022	Retrospective	Unicenter	Japan	Acute cholecystitis	20	EGBS	Nasobiliary drainage tube/Pigtail stent		92.3% (12/13) cut ENGBD tube stent; 100% (7/7) ready-made double-pigtail stent	7.7% (1/13) cut ENGBD tube stent; 0% (0/7) ready-made double-pigtail stent		Median observation: 273 d (IQR 28–1579) cut ENGBD tube stent; 628 d (IQR 389–1070) ready-made double-pigtail stent	Measure and cut the pigtail ENGBD tube and place the newly created stent/place a ready-made double-pigtail stent
Maruta et al. [49]	2022	Retrospective	Multicenter	Japan	Acute cholecystitis	21	EGBS	Nasobiliary drainage tube	90.5% (19/21)	100% (19/19)	Procedural 9.5% (2/21); late 4.7% (1/21)	0% (0/19)	Median 61 d (19–103) to surgery	Cut the ENGBD tube with a loop cutter in conjunction with surgical scissors inside the stomach
Ban et al. [50]	2023	Case report		Japan	Acute cholecystitis with cholelithiasis and choledocholithiasis	1	EGBS	Drilling dilator/Double-pigtail plastic stent	100% (1/1)					Aid plastic stent delivery with a screw tip stent retriever
Sato et al. [51]	2023	Retrospective	Unicenter	Japan	Acute cholecystitis	242	ENGBD/EGBS	Plastic stent or nasobiliary drainage tube	86.8% (210/242)	92.9% (195/210)	9.9% (24/242) CD injury; 5.0% (12/242) ERCP-related AEs			
Ishii et al. [52]	2023	Retrospective	Unicenter	Japan	Cholecystitis after covered metal stent placement for distal biliary obstruction	32	EGBS	Double-pigtail plastic stent	75.0% (24/32)		EGBS-related AE 9.4% (3/32)	Post-CSEMS AC incidence 4.2% (1/24) EGBS; 21.4% (56/262) no EGBS	Median 175 d	Place prophylactic endoscopic gallbladder stent before the CSEMS deployment
Inoue et al. [12]	2023	Prospective	Unicenter	Japan	Acute calculous cholecystitis	83	EGBS	Single-pigtail stent	80.7% (67/83)	98.5% (66/67)	Early 3.6% (3/83); overall late 10.6% (7/66)	4.5% (3/66)	Median 603 d (IQR 404–1314)	Perform endoscopic gallbladder inside-stenting with lavage and aspiration
Koizumi et al. [53]	2023	Case report		Japan	Acute cholecystitis	2	ENGBD/EGBS	Nasobiliary drainage tube/Pigtail stent	100% (2/2)					Place the tube or stent through the outer sheath into the fundus of the gallbladder
Yoshida et al. [21]	2023	Retrospective	Unicenter	Japan	Acute cholecystitis	115	ENGBD/EGBS	Three-pillar: cholangioscopy, flex-type guidewire, 3-Fr microcatheter	72.0% (36/50) classical; 96.9% (63/65) strategic	91.7% (33/36) classical; 93.7% (59/63) strategic	12.0% (6/50) classical; 10.8% (7/65) strategic			Strategic ETGBD/classical ETGBD
Maruta et al. [54]	2023	Retrospective	Multicenter	Japan	Acute cholecystitis	122	EGBS	Nasobiliary drainage tube	78.6% (96/122) ENGBD; 91.1% (31/34) cutting	96.8% (93/96) ENGBD improvement	Early 3.2% (4/122); cutting procedural AE 8.8% (3/34); late AE 6.4% (2/31) cutting, 33.3% (12/36) removal	6.4% (2/31) cutting; 11.1% (4/36) removal	Median waiting 58 d (IQR 43–80) cutting; 33 d (IQR 14–78) removal	Cut the ENGBD tube with a loop cutter in conjunction with surgical scissors inside the stomach
Hirakawa et al. [19]	2024	Retrospective	Unicenter	Japan	Acute cholecystitis	182	ENGBD/EGBS	Nasobiliary drainage tube/Double-pigtail stent	84.6% (154/182)	96.1% (148/154)	11.0% (20/182)			
Noda et al. [55]	2024	Case report		Japan	Acute cholecystitis and cholangitis	1	EGBS	Drilling dilator/Plastic stent	100% (1/1)		0% (0/1)			Insert a novel drill-type dilator and perform EGBS
Suzuki et al. [10]	2024	Retrospective	Unicenter	Japan	Acute cholecystitis	71	ENGBD	Nasobiliary drainage tube	85.9% (61/71)	93.4% (57/61)	11.3% (8/71)			Place the nasobiliary catheter or cut the tube within the stomach
Niiya et al. [20]	2024	Retrospective	Unicenter	Japan	Acute cholecystitis	35	EGBS	Plastic stent	77.1% (27/35)		Early 11.4% (4/35); late 3.7% (1/27)	3.7% (1/27)	Median 123 d (20–1435)	Perform EGBS after PTGBD placement
Ishikawa et al. [56]	2025	Case report		Japan	Acute cholecystitis	1	EGBS	Novel rotatable sphincterotome/IYO stent	100% (1/1)	100% (1/1)	0% (0/1)			Rotatable sphincterotome for difficult caudal cystic duct cannulation
Cooper et al. [57]	2025	Retrospective	Unicenter	USA	Symptomatic cholelithiasis/biliary disease in decompensated liver disease	55	EGBS	Pigtail stent	94.5% (52/55)	92.3% (48/52)	7.7% (4/52) 30-d AE; 1.9% (1/52) 30-d mortality	7.7% (4/52) at 6 mo	Median 361 d (IQR 117–766)	
Goda et al. [58]	2025	Case report		Japan	Acute cholecystitis	1	ENGBD	Rotatable Engetsu catheter/Nasobiliary drainage tube	100% (1/1)	100% (1/1)	0% (0/1)			Rotatable catheter for difficult stricture alignment and guidewire passage
Kuraishi et al. [59]	2025	Case report		Japan	Acute cholecystitis	1	EGBS	Pigtail stent	100% (1/1)					Loop cutter-assisted over-the-wire stent exchange
Nakahara et al. [60]	2026	Retrospective	Unicenter	Japan	Acute cholecystitis	24	EGBS	Novel plastic stent with an integrated delivery system	95.8% (23/24)	94.7% (18/19) EGBS; 90.0% (18/20) overall	Early 4.2% (1/24); late 10.0% (2/20)	0% (0/20)	Mean 211 d	

Abbreviations: AC, acute cholecystitis; AE, adverse event; AEs, adverse events; ATT, antithrombotic therapy; CD, cystic duct; CDP, cystic duct perforation; CSEMS, covered self-expandable metal stent; EGBS, endoscopic gallbladder stenting; ENGBD, endoscopic nasogallbladder drainage; ETGBD, endoscopic transpapillary gallbladder drainage; ERCP, endoscopic retrograde cholangiopancreatography; EUS-GBS, endoscopic ultrasound-guided gallbladder stenting; IDUS, intraductal ultrasonography; IQR, interquartile range; LC, laparoscopic cholecystectomy; PTGBD, percutaneous transhepatic gallbladder drainage; SEMS, self-expandable metal stent; SOC, single-operator cholangioscopy; SpyDS, SpyGlass Direct Visualization System; d, days; mo, months; y, years.

**Table 2:** Practical interpretation of the four-step classification for ETGBD.

Step	Main Technical Objective	Common Signals of Difficulty or Failure	Potential Adjunctive or Rescue Strategies
Step 1	Identify the cystic duct orifice and define the biliary–cystic duct anatomy	Poor cystic duct opacification, non-visualization of the cystic duct, or unclear cystic duct takeoff	Repeat cholangiography, balloon-occlusion cholangiography, intraductal ultrasonography, cholangioscopy-assisted identification, or PTGBD-assisted staged approach
Step 2	Obtain guidewire access into the cystic duct	Failure to enter the cystic duct due to steep takeoff angle, tortuosity, stenosis, stone impaction, or unfavorable cystic duct orientation	Hydrophilic or smaller-caliber guidewire, rotatable catheter, balloon-occlusion method, cholangioscopy-assisted cannulation
Step 3	Advance and stabilize the guidewire within the gallbladder	Loss of guidewire support, looped guidewire formation, inability to cross true obstruction, or failure to achieve stable gallbladder access	Microcatheter assistance, double-guidewire technique, balloon catheter straightening, cholangioscopy-guided passage, or alternative drainage if obstruction cannot be crossed
Step 4	Deliver and maintain the drainage catheter or stent	Failed device advancement, mismatch in stent length or shape, inadequate guidewire support, early migration, occlusion, or recurrent cholecystitis	Appropriate stent selection, dedicated gallbladder stents, dilators or delivery-assisting devices, dual-stent placement, staged stent exchange, or alternative drainage

Abbreviations: ETGBD, endoscopic transpapillary gallbladder drainage; PTGBD, percutaneous transhepatic gallbladder drainage.

**Table 3:** Clinical comparison of gallbladder drainage strategies for acute cholecystitis.

**Drainage Method**	**Author**	**Year**	**Format**	**Scale**	**Country**	**Disease**	Patients	Drainage Procedure	Drainage Device	Technical Success	Clinical Success	Adverse Events	Recurrence/Late Outcomes	Recurrence Definition/Group	Follow-Up Duration
ETGBD vs. PC	Inoue et al. [62]	2016	Retrospective	Unicenter	Japan	Acute calculous cholecystitis	35	EGBS	Plastic stent	82.9% (29/35)		Procedure 2.9% (1/35); late non-AC 9.1% (3/33)	0% (0/33)	Recurrent cholecystitis; EGBS group	Median 473 d (13–1095)
							29	PTGBD-rendezvous method with second EGBS	Pigtail tube	100% (29/29)		Late non-AC 6.9% (2/29)	17.2% (5/29)	Recurrent cholecystitis; PTGBD group	Median 485 d (30–1873)
	Itoi et al. [91]	2017	Retrospective	Multicenter	Japan	Acute cholecystitis	330	ENGBD/EGBS	Single-pigtail nasogallbladder catheter/Double-pigtail stent		89.2% (207/232)	8.2% (27/330)			
							266	PTGBD	Pigtail catheter		85.7% (191/223)	5.6% (15/266)			
							64	PTGBA	Aspiration needle with optional saline lavage		96.2% (50/52)	1.6% (1/64)			
	Iino et al. [90]	2018	Retrospective	Unicenter	Japan	Acute cholecystitis	43	ENGBD/EGBS	Nasobiliary drainage tube/Double-pigtail stent	77.0% (33/43)		9.3% (4/43)			
							42	PTGBD	Pigtail catheter	100% (42/42)		4.8% (2/42)			
	Kaura et al. [89]	2020	Retrospective	Unicenter	USA	Acute cholecystitis	57	EGBS	Double-pigtail plastic stent	91.2% (52/57)		Unplanned repeat intervention 7.7% (4/52); postoperative complications 30.8% (16/52)	Clinical failure/recurrent AC 7.7% (4/52)	Clinical failure and recurrence of cholecystitis; ERGD successful patients	Median 61 d (IQR 32–98) from drainage to cholecystectomy
							140	PC	Pigtail catheter	100% (140/140)		Unplanned repeat intervention 16.4% (23/140); postoperative complications 47.1% (66/140)		AC recurrence not separately reported; unplanned repeats were drain blockage/dislodgement	Median 72 d (IQR 51–109) from drainage to cholecystectomy
	Mu et al. [92]	2021	Prospective	Unicenter	China	Acute cholecystitis	11	ENGBD	Nasobiliary drainage tube/Double-pigtail stent	90.9% (10/11)	100% (11/11)	Peri-drainage 9.1% (1/11); peri-cholecystectomy 0% (0/11)	9.1% (1/11)	Recurrent cholecystitis; randomized group	Bridge to cholecystectomy 2–3 mo; follow-up ≥3 mo after cholecystectomy
							11	PTGBD	Pigtail catheter	100% (11/11)	100% (11/11)	Peri-drainage 27.3% (3/11); peri-cholecystectomy 0% (0/11)	0% (0/11)	Recurrent cholecystitis; randomized group	Bridge to cholecystectomy 2–3 mo; follow-up ≥3 mo after cholecystectomy
	Inoue et al. [94]	2023	Retrospective	Multicenter	Japan	Acute calculous cholecystitis	297	EGBS	Double-pigtail stent/Single-pigtail stent	75.4% (224/297)	96.9% (217/224)	Early 7.7% (23/297); late non-AC symptomatic 19.6% (31/158); overall late 25.9% (41/158)	6.3% (10/158)	Recurrent cholecystitis; long-term EGBS patients	Mean 1115.5 d (SD 729.2)
							231	PTGBD	Pigtail catheter	98.7% (228/231)	96.9% (221/228)	Early 4.3% (10/231); late non-AC symptomatic 10.8% (13/120); overall late 30.0% (36/120)	19.2% (23/120)	Recurrent cholecystitis; long-term PTGBD patients	Mean 1136.2 d (SD 728.8)
ETGBD vs. EUS-GBD	Oh et al. [95]	2019	Retrospective	Unicenter	Korea	Acute cholecystitis	96	EGBS	Double-pigtail plastic stent	83.3% (80/96)	82.3% (79/96)	Procedural AE 9.4% (9/96); asymptomatic stent-related AE 4.3% (3/69)	Cholecystitis/cholangitis recurrence 17.4% (12/69); recurrent cholecystitis 14.5% (10/69)	Recurrent typical symptoms with characteristic imaging findings; long-term patients	Mean 25.5 mo (SD 21.9)
							83	EUS-GBD	SEMS	98.8% (82/83)	98.8% (82/83)	Procedural 7.2% (6/83); asymptomatic stent-related 3.9% (3/76)	Cholecystitis/cholangitis recurrence 3.9% (3/76); recurrent cholecystitis 3.9% (3/76)	Recurrent typical symptoms with characteristic imaging findings; long-term patients	Mean 18.6 mo (SD 21.1)
	Higa et al. [96]	2019	Retrospective	Unicenter	USA	Acute cholecystitis	38	EGBS	Double-pigtail stent	84.2% (32/38)	76.3% (29/38)	9.4% (3/32)	18.8% (6/32)	Recurrent cholecystitis during follow-up; successful transpapillary drainage patients	Median 5 mo (<1–29)
							40	EUS-GBD	LAMS	97.5% (39/40)	95.0% (38/40)	17.9% (7/39)	2.6% (1/39)	Recurrent cholecystitis during follow-up; successful EUS drainage patients	Median 7 mo (<1–20)
	Nishiguchi et al. [97]	2021	Retrospective	Unicenter	Japan	Acute cholecystitis	29	EGBS with or without digital single-operator cholangioscope	Plastic stent	89.7% (26/29) overall	79.3% (23/29)	Procedure-related 10.3% (3/29); stent-related 7.7% (2/26)	15.4% (4/26)	Recurrent cholecystitis; successful ETGBD patients	Median follow-up reported as 522 d (range 43–1892)
							25	EUS-GBD	FCSEMS plus double-pigtail plastic stent	100% (25/25)	96.0% (24/25)	Procedure-related 4.0% (1/25); stent-related 0% (0/25)	0% (0/25)	Recurrent cholecystitis; EUS-GBD patients	Median follow-up reported as 522 d (range 43–1892)
	Inoue et al. [98]	2023	Retrospective	Multicenter	Japan	Acute calculous cholecystitis	90	EGBS	Double-pigtail stent/Single-pigtail stent	78.9% (71/90)	94.4% (67/71)	Early 8.9% (8/90); late non-AC symptomatic 13.4% (9/67); overall late 16.4% (11/67)	3.0% (2/67)	Recurrent cholecystitis; clinical-success ETGBD patients	Mean 689.8 d (SD 530.8) after PSM
							90	EUS-GBD	Double-pigtail plastic stent/Nasobiliary drainage tube	96.7% (87/90)	92.0% (80/87)	Early 7.8% (7/90); late non-AC symptomatic 1.3% (1/80); overall late 5.0% (4/80)	3.8% (3/80)	Recurrent cholecystitis; clinical-success EUS-GBD patients	Mean 727.6 d (SD 411.7) after PSM
	Amin et al. [78]	2025	Retrospective	Multicenter	USA	Acute cholecystitis	73	EGBS	Dual transcystic stents	ETGBD 86.3% (63/73); DSET 80.9% (51/63)	100% (36/36) for index DSET-GBD	Procedure-related 0% (0/51); 30-d mortality 2.0% (1/51)	5.3% (2/38)	Recurrent cholecystitis; patients with non-cholecystitis death during follow-up excluded from primary outcome	Minimum 6 mo; primary recurrence assessed within 6 mo
							56	EUS-GBD	LAMS	87.5% (49/56)	98.1% (52/53)	Procedure-related 2.0% (1/49); 30-d mortality 12.2% (6/49)	8.2% (4/49)	Recurrent cholecystitis; all patients who eventually had stents placed, excluding non-evaluable patients	Minimum 6 mo; primary recurrence assessed within 6 mo
ETGBD vs. PC vs. EUS-GBD	Siddiqui et al. [82]	2019	Retrospective	Multicenter	International	Acute cholecystitis	124	EGBS	Double-pigtail plastic stent	87.9% (109/124)	79.8% (99/124)	Procedural 7.3% (9/124); long-term 4.8% (6/124); unplanned admission 3.2% (4/124)	3.2% (4/124)	Recurrent cholecystitis; all EGBS patients	Median 3 mo (IQR 3–6)
							102	EUS-GBD	LAMS	94.1% (96/102)	90.2% (92/102)	Procedural 11.8% (12/102); long-term 1.9% (2/102); unplanned admission 3.9% (4/102)	1.0% (1/102)	Recurrent cholecystitis; all EUS-GBD patients	Median 4 mo (IQR 3–9)
							146	PTGBD	Pigtail drainage catheter	97.9% (143/146)	96.6% (141/146)	Procedural 4.1% (6/146); long-term 19.9% (29/146); unplanned admission 19.9% (29/146)	2.7% (4/146)	Recurrent cholecystitis; all PTGBD patients	Median 3 mo (IQR 3–4)
	Ridtitid et al. [99]	2022	Retrospective	Unicenter	Thailand	Acute cholecystitis with or without concomitant common bile duct stones	93	EGBS either by fluoroscopic or digital cholangioscopic guidance	Double-pigtail plastic stent	74.2% (69/93)	95.7% (66/69)	10.5% (9/86)	4.6% (4/86)	Recurrent clinical symptoms plus imaging findings of AC; final EGBS-group patients	Subgroup medians 193 d with CBD stones and 197 d without CBD stones
							22	EUS-GBD	LAMS/FCSEMS	100% (22/22)	100% (22/22)	4.5% (1/22)	4.5% (1/22)	Recurrent clinical symptoms plus imaging findings of AC; final EUS-GBD-group patients	Subgroup medians 252 d with CBD stones and 390 d without CBD stones
							35	PC	Pigtail drainage catheter	100% (35/35)	68.6% (24/35)	0% (0/35)	17.1% (6/35)	Recurrent clinical symptoms plus imaging findings of AC; PC-group patients	Subgroup medians 79 d with CBD stones and 72 d without CBD stones

Abbreviations: AC, acute cholecystitis; AEs, adverse events; CBD, common bile duct; DSET, dual-stent endoscopic transpapillary gallbladder drainage; EGBS, endoscopic gallbladder stenting; ENGBD, endoscopic nasogallbladder drainage; ERGD, endoscopic retrograde gallbladder drainage; ETGBD, endoscopic transpapillary gallbladder drainage; EUS-GBD, endoscopic ultrasound-guided gallbladder drainage; FCSEMS, fully covered self-expandable metal stent; IQR, interquartile range; LAMS, lumen-apposing metal stent; PC, percutaneous cholecystostomy; PTGBA, percutaneous transhepatic gallbladder aspiration; PTGBD, percutaneous transhepatic gallbladder drainage; PSM, propensity score matching; SD, standard deviation; SEMS, self-expandable metal stent; TP, transpapillary; d, days; mo, months.

## Data Availability

No new data were created or analyzed in this study. Data sharing is not applicable to this article.

## Supplementary Materials

The following supporting information can be downloaded at: https://www.mdpi.com/article/10.3390/jcm15156007/s1, Table S1: Detailed evidence extraction of ETGBD-related studies; Table S2: Detailed evidence extraction of comparative studies involving ETGBD, PTGBD, and EUS-GBD.

## Abbreviations

The following abbreviations are used in this manuscript:

AC	Acute Cholecystitis
LC	Laparoscopic Cholecystectomy
PTGBD	Percutaneous Transhepatic Gallbladder Drainage
ETGBD	Endoscopic Transpapillary Gallbladder Drainage
EUS	Endoscopic Ultrasound
EUS-GBD	Endoscopic Ultrasound-Guided Gallbladder Drainage
ERCP	Endoscopic Retrograde Cholangiopancreatography
ENGBD	Endoscopic Nasogallbladder Drainage
EGBS	Endoscopic Gallbladder Stenting
CD	Cystic Duct
GW	Guidewire
IDUS	Intraductal Ultrasonography
SpyDS	SpyGlass Direct Visualization System
LAMS	Lumen-Apposing Metal Stent
SEMS	Self-Expanding Metallic Stent
AE	Adverse Event
CDP	Cystic Duct Perforation
ATT	Antithrombotic Therapy
